# Plant Trait-Species Abundance Relationships Vary with Environmental Properties in Subtropical Forests in Eastern China

**DOI:** 10.1371/journal.pone.0061113

**Published:** 2013-04-03

**Authors:** En-Rong Yan, Xiao-Dong Yang, Scott X. Chang, Xi-Hua Wang

**Affiliations:** 1 Department of Environmental Sciences, East China Normal University, Shanghai, China; 2 Tiantong National Station of Forest Ecosystem, Chinese National Ecosystem Observation and Research Network, Ningbo, China; 3 Department of Renewable Resources, University of Alberta, Edmonton, Canada; Duke University, United States of America

## Abstract

Understanding how plant trait-species abundance relationships change with a range of single and multivariate environmental properties is crucial for explaining species abundance and rarity. In this study, the abundance of 94 woody plant species was examined and related to 15 plant leaf and wood traits at both local and landscape scales involving 31 plots in subtropical forests in eastern China. Further, plant trait-species abundance relationships were related to a range of single and multivariate (PCA axes) environmental properties such as air humidity, soil moisture content, soil temperature, soil pH, and soil organic matter, nitrogen (N) and phosphorus (P) contents. At the landscape scale, plant maximum height, and twig and stem wood densities were positively correlated, whereas mean leaf area (MLA), leaf N concentration (LN), and total leaf area per twig size (TLA) were negatively correlated with species abundance. At the plot scale, plant maximum height, leaf and twig dry matter contents, twig and stem wood densities were positively correlated, but MLA, specific leaf area, LN, leaf P concentration and TLA were negatively correlated with species abundance. Plant trait-species abundance relationships shifted over the range of seven single environmental properties and along multivariate environmental axes in a similar way. In conclusion, strong relationships between plant traits and species abundance existed among and within communities. Significant shifts in plant trait-species abundance relationships in a range of environmental properties suggest strong environmental filtering processes that influence species abundance and rarity in the studied subtropical forests.

## Introduction

Understanding factors affecting the distribution of plant species along environmental gradients is one of the central focuses in ecology. Ecologists have long tried to explain why some species are more abundant than others across habitats [1]–[7]. Mechanisms influencing the presence and abundance of species in a community may include the biogeographic history of the region, environmental conditions, and stochastic processes [8], [9]. In addition, species attributes such as dispersal ability, physiological tolerances and associated trait-based niche-assembly influence the structure of the community [10]–[20].

Trait-based niche-assembly demonstrates that measurable traits of plant species play a crucial role for the success or failure of a species, which is usually measured as the presence? absence of species in specific environmental conditions [21]. There is emerging evidence showing trait-based patterns of species presence or absence across communities [19], [20], [22]–[30]. All of these empirical studies suggest that plant distribution patterns along environmental gradients reflect a functional trait-based principle that structures ecological communities, because the distribution of species is expected to be determined by the availability of resources. The functional strategy of a species will dictate its resource use and therefore its location along a resource axis or axes. Therefore, the variability of plant trait-species abundance relationships along a range of environmental properties should be predictable.

Understanding how plant trait-species abundance relationships change along a range of environmental properties is vital for predicting the effects of specific environmental factors on species abundance and rarity. Here we present an account of putative mechanisms to test alternative hypotheses regarding how the potential plant trait-species abundance relationship varies with a range of environmental properties. In particular, we focus on air humidity, soil moisture content, soil temperature, soil fertility and pH, because they are basic abiotic factors that influence resource use strategies of plants [31], [32].

It has been recognized that habitat conditions in a specific site can form an environmental barrier to affect species establishment and/or survival [15], [22], [25]. The success of species in this condition depends on its maximum tolerance to physiological and morphological attributes [13], [17], [19], [22], [23], [25], [27], [28], [33]. In this case, environmental conditions are assumed to drive the optimal trait combinations, which then select for the appropriate species mix [22], [34], [35] that eventually affects species abundance. For example, plants with relatively small size leaves benefit more from dry and nutrient-poor habitats, while wet, shady, and fertile habitats tend to favor plants with relatively rapid growth, and large leaves [19], [36], [37].

Based on our understanding of relationships between plant growth and environment conditions, we make the following four predictions: 1) in wet habitats with high soil moisture availability to roots [30], [38], the most abundant species will have larger leaves and lower leaf dry matter content (LDMC, leaf dry mass per leaf fresh mass) than those in dry habitats; 2) as a result of decreasing air humidity in warm habitats [36], [37], the most abundant species in such habitats will have higher LDMC, lower leaf area and leaf N concentration than those in cooler habitats; 3) the most abundant plant species in infertile habitats are expected to have low foliar nutrient concentrations and high LDMC [39]–[40]; and thus high wood density due to slow growth rates and long nutrient residence times [39]; and 4) the most abundant species in acidic habitats will have higher leaf area than those in habitats with neutral soil pH, because acidic soils in the studied subtropical forests are usually related to the late-successional status with high soil nutrient availability [41].

We tested these four predictions in subtropical forests in eastern China. Specifically, we were interested in understanding 1) whether plant leaf and wood traits are associated with species abundance; and 2) how environmental properties such as soil moisture, temperature, nutrient content and soil pH shape plant trait-species abundance relationships.

## Materials and Methods

### Ethics Statement

No specific permits were required for the described field studies in and outside of Tiantong Forest Park (TT), Ruiyan Forest Park (RY), and Dongqian Lake Landscape Area (DQ). These three forest parks are owned and managed by the local government and the location including the site for our sampling are not privately-owned or protected in any way and thus a specific permit for not-for--profit research is not required. The field studies did not involve endangered or protected plant species in this area.

### Study area, vegetation, environmental ranges and sampling plots

This study was conducted in the lower eastern extension of the Siming Mountain (29°41–50′N, 121°36–52′E), Zhejiang province, in eastern China. The region has a typical monsoon climate with a hot humid summer and a dry cold winter. The highest peak in this area is at 653 m above sea level, while most other reliefs are in the 70–300 m range [42].

The zonal vegetation in this region is subtropical evergreen broadleaf forests (EBLFs), which have been severely disturbed in recent history with only small tracts of intact or semi-intact EBLFs left around a Buddhist temple in the Tiantong Forest Park (TT), as well as in Ruiyan Forest Park (RY) and in Dongqian Lake Landscape Area (DQ). These three sites, spaced approximately 15 km from each other, had been subjected to different intensities of human disturbances (usually logging), but have been protected from logging and clearcutting from 25 yrs ago. Consequently, the integral plant community structure was different among the three sites. The vegetation was characterized by three vertical layers (tree, sub-tree and shrub) in TT, two layers (tree and shrub) in RY, and a shrub layer only in DQ. Thus, the vegetation in those three sites represented different successional status, with vegetation being mature and intact in TT, semi-mature in RY, and young (i.e., shrubs) in DQ [41]. In addition, among the three sites, TT had the largest area of EBLFs with a relatively wider range of topographic features, including a mesophytic area (TM), a peak area (TP), and a ravine area (TR). In general, plant species compositions in these three sites were similar. Local micro-environmental conditions differed with the different terrain features, the EBLFs in TM, TP, TR, RY and DQ represented a topography-mediated range of soil properties in the region.

The EBLFs in each of the five sites (TM, TP, TR, RY and DQ) were selected to represent a range of environmental properties. Plots within each of those sites were systematically established 100–500 m apart, located on the same slope position and having similar vegetation history and soil type (i.e., mainly Red and Yellow soils in the Chinese system of soil classification) and soil texture (i.e., mainly loam textures). We established 31 plots in total, covering all typical habitats in this region, with nine in TM, four in TP, six in TR, four in RY, and eight in DQ. Each plot (20×20 m) was located at least 100 m away from the stand edge. Because the 20×20 m plot is the smallest reasonable area to be considered as a community for subtropical forests [42], we used the plot to represent a forest community with a set of species coexisting and interacting in a locality.

### Data collection

#### Plant traits

For leaf, twig and stem traits, at least three plants per species were randomly selected and marked in each plot, and then leaf, twig and stem samples were collected in July and August 2008. We measured 15 plant traits for each sampled individual, including LDMC, specific leaf area (SLA, leaf area divided by dry mass), mean leaf area (MLA), leaf N and P concentrations in both mature (from one year old or older twigs) and current year leaves, total leaf area carried on current year twigs (TLA), twig dry matter content (TDMC, dry mass divided by fresh mass), twig wood density (TWD, dry mass divided by volume), stem wood density (SWD), and plant height. We used the maximum plant height of each species that occurred in the 31 plots to represent potential plant vertical growth as their ability for light interception.

In the field, five branches were cut from five different positions in each sampled plant, i.e., from the four sides and the upper position of the crown. Since leaf longevity significantly affects plant nutrient use strategies [31], [33], here we measured leaf traits in both mature and current year leaves to test whether any potential plant trait-species abundance relationship differ with leaf age. We separated the current year twigs from the collected branches according to the terminal set of internodes. From each branch, approximately 20 mature leaves without apparent leaf damage were chosen and sampled, and then one current year twig without apparent leaf loss was sampled. Each sample was wrapped in a moist paper towel and stored in a sealed plastic bag and kept cool until brought back to the laboratory for measurement.

In the laboratory, the leaves were separated from the twig, then twig length and twig diameter at the mid point were measured. Twig diameter was measured using an electronic vernier caliper (accurate to 0.1 mm). Twig cross-sectional area was calculated from the diameter. Twig volume was assumed to be approximate to a cylinder shape, with mid-point stem diameter as the cylinder diameter and stem length as the cylinder length. At the same time, 20 mature leaves from each branch and all fresh leaves attached on the current year twig were collected to form separate samples. The leaves in those samples were then scanned using a leaf area meter (LI-3100C, Li-Cor, USA) to determine the MLA for each mature and current year leaf, and TLA of the current year twigs. Then twig and leaf samples were dried at 75°C for 48 hrs in an oven to determine twig and leaf dry mass, for calculating TDMC, LDMC, SLA and TWD. Finally, the leaf samples were ground to determine leaf N and P concentrations using a flow-injection auto-analyser (Skalar, Netherland).

We used tree increment cores to determine stem wood density. Using a 5 mm-diameter increment corer, we collected one core at breast height from each plant that was used for collecting twig and leaf samples. In the laboratory, the length of each tree core was measured using an electronic vernier caliper (accurate to 0.1 mm) to determine the diameter of the tree, then the volume of each tree core was calculated, assuming the tree increment core was approximate to a cylinder shape. Samples were then dried at 75°C in an oven for 72 hrs to determine dry mass and to calculate SWD.

#### Environmental parameters

Air humidity and soil moisture content in October were used to represent a site's moisture regime, soil temperature was used to represent the thermal regime, soil organic matter along with N and P contents were used to represent soil fertility, and soil pH was measured to represent soil reaction. Air humidity was examined for two weeks using three Hobo Air Humidity Smart Sensors (Japan) installed 1.5 m aboveground within each plot in October 2008. At the same time, soil moisture content and temperature in the 0-20 cm soil were measured using three HOBO Soil Moisture and Temperature Smart Sensors (Japan) in each plot. Although two weeks were not enough to measure meaningful differences among sites for these external variables, it could reflect potential variation in air humidity, soil moisture and temperature among plots. The studied plots were located very close to each other and thus in general received the same amount of rainfall [39].

In addition, five soil samples (0-20 cm soil layer) were taken with a metal corer from randomly chosen positions in each plot, resulting in 155 samples (5 samples per plot ×31 plots). Soil samples were air-dried for 30 days. A subsample from each of the 155 soil samples was passed through a 0.5 mm sieve, and approximate 10 g in each subsample was digested to determine total N and P concentrations using a flow-injection autoanalyser (Skalar, Netherland). Another subsample from each of the 155 soil samples was ground to pass a 2 mm sieve to determine soil organic matter concentration using the oil bath-K_2_CrO_7_ titration method. A third subsample was used to measure soil pH in water at a 1:2 ratio (w:v for soil vs water) using a Metterler-143 Toledo pH meter.

### Data analysis

#### Plant trait-species abundance relationship

On average, stem density of woody species with DBH (diameter at breast height) ≥1 cm was 36 per plot. In each plot, species composition, plant height, and stem basal area (based on diameter measured at 5 cm from ground for seedlings and saplings, and at 20 cm from ground for trees) for all individuals with a height >0.2 m were measured. We used the number of individuals, total stem basal area of a given species, and plot frequency (the number of plots in which a species was observed) to represent species abundance at the landscape scale. At the plot scale, only the first two indices were used. Here, we focused on the number of individuals with seedlings included. Seedlings were often neglected in previous studies [19], leading to a weak relationship between plant traits and species abundance [43].

Plant trait-species abundance relationships were analysed at both the landscape and plot scales. At the landscape scale, we conducted three analyses to estimate species abundance. In the first two procedures, we summed the number of individuals and the basal area for each species in 31 plots to represent the abundance for each of the 94 species. Then each of these two abundance indices was correlated respectively with the mean trait values of each of the 15 plant traits for the 94 species. In the last procedure we counted the number of plots in which a species was observed and then we correlated the frequency of observation (i.e., number of plots) with the mean trait values for that species.

At the plot scale, the number of individuals and the total basal area for each species occurring in a given plot were used to estimate species abundance. Pearson correlation analysis was used to relate the mean trait values in each of the 15 plant traits to the two abundance indices for each species in each plot. In total, 31 r values for each trait were generated to result in a median value of r. We then used a one-sample Wilcoxon test to determine whether the median of the r-values was statistically different from zero. If the null hypothesis was correct, and the mean of this distribution was statistically not different from zero, then there was no relationship between the trait value of a species and abundance; otherwise, there was a significant relationship between plant trait and species abundance [44].

#### Shift patterns of plant trait- species abundance relationships along a range of environmental properties

To test whether any potential plant trait-species abundance relationships were affected by the seven environmental properties, the 31 Pearson correlation coefficient r values that were generated at the plot scale for each trait in the trait-species abundance relationship were correlated separately with each of air humidity, soil moisture content, soil temperature, soil pH, and soil organic matter, N and P contents. A significant relationship between an environmental property and the r-value of a trait-abundance relationship showed that the strength of the correlation between plant trait and species abundance was shifting across the range of environmental property. This is a result that describes every significant relationship between the r-value of a trait-abundance relationship and an environmental property. On this basis, a significant direction shift of a trait-abundance relationship across a range of environmental measurement emerged only when the direction of a trait-abundance relationship reversed across an environmental property. The reversed direction was defined here as the change of correlation coefficients of the trait-abundance relationship from positive to negative (or vice/versa) across the range of values of the environmental property [19]. If the correlation coefficients remain positive or negative in the whole range of the environmental property, this means that there was no significant direction reversal in a plant trait-species abundance relationship.

In addition, we conducted a principle component analysis (PCA, using a correlation matrix) on seven environmental properties to identify major multivariate ranges, and then explored how any potential plant trait-species abundance relationships change along these major axes. In this case, we assumed that the principle component receiving the highest eigenvalues best represents the variation in the environmental properties. Therefore, only the first two principle components with eigenvalues ≥1, loading more than 66% of the total variation in habitat properties (see Results), were extracted to characterize major multivariate gradients for all subsequent analyses. After this, the 31 r values generated from the plot scale were correlated to the scores generated from the first two principal components.

## Results

### Relationships between plant traits and species abundance

Plant trait-species abundance relationships showed contrasting patterns between the landscape and plot scales and among three abundance measurements (Table 1). At the landscape scale, plant maximum height, TWD and SWD were positively correlated, while MLA and leaf N concentrations in both mature and current year leaves, and TLA were negatively correlated with both the number of individuals and plot frequency. However, LDMC, SLA, and leaf P concentrations in both mature and current year leaves along with TDMC were not significantly correlated with these two abundance indices. For basal area, positive relationships were only found for plant maximum height and TWD (Table 1).

**Table 1:** Summary of correlation coefficients in the relationship between 15 plant functional traits and species abundance.

	Landscape scale						Plot scale			
	Abundance		Total basal area		Plot frequency		Abundance		Total basal area	
	r**^‡^**	p^‡^	r	p	r	p	Median r	Wilcoxon	Median r	Wilcoxon
MH†	**0.44**	**<0.001**	**0.75**	**<0.001**	**0.47**	**<0.001**	**0.43**	**<0.001**	**0.77**	**<0.001**
LDMC-M	0.12	NS	0.18	NS	0.12	NS	**0.21**	**<0.01**	**0.25**	**<0.001**
LDMC-F	0.16	NS	0.11	NS	0.17	NS	**0.13**	**<0.001**	**0.21**	**<0.001**
LA-M	**−0.28**	**<0.01**	**−**0.08	NS	**−0.28**	**<0.01**	**−0.20**	**<0.001**	0.06	NS
LA-F	**−0.36**	**<0.001**	**−**0.18	NS	**−0.33**	**<0.001**	**−0.17**	**<0.001**	**−**0.003	NS
TLA	**−0.21**	**<0.05**	**−**0.02	NS	**−0.21**	**<0.05**	**−0.30**	**<0.001**	**−**0.01	NS
SLA-M	**−**0.09	NS	**−**0.09	NS	**−**0.12	NS	**−0.23**	**<0.001**	**−0.15**	**<0.01**
SLA-F	**−**0.12	NS	**−**0.07	NS	**−**0.13	NS	**−0.25**	**<0.001**	**−0.26**	**<0.01**
N_mass_-M	**−0.23**	**<0.05**	**−**0.17	NS	**−0.41**	**<0.001**	**−0.33**	**<0.01**	**−**0.04	NS
N_mass_-F	**−0.23**	**<0.05**	**−**0.17	NS	**−0.33**	**<0.01**	**−0.34**	**<0.001**	**−0.12**	**<0.05**
P_mass_-M	0.06	NS	0.19	NS	0.09	NS	**−**0.14	NS	-0.03	NS
P_mass_-F	**−**0.10	NS	**−**0.03	NS	**−**0.13	NS	**−0.12**	**<0.05**	**−**0.18	NS
TDMC	0.09	NS	−0.05	NS	0.06	NS	**0.13**	**<0.05**	0.03	NS
TWD	**0.30**	**<0.01**	**0.25**	**<0.05**	**0.32**	**<0.01**	**0.12**	**<0.05**	**0.10**	**<0.05**
SWD	**0.31**	**<0.01**	0.11	NS	**0.27**	**<0.05**	**0.11**	**<0.05**	−0.03	NS

† : MH: Plant maximum height; LDMC-M: Leaf dry matter content of mature leaves; LDMC-F: Leaf dry matter content of current year's leaves; LA-M: Mean leaf area of mature leaves; LA-F: Mean leaf area of the current year's twigs; TLA: Total leaf area attached on the current year's twigs; SLA-M: Specific leaf area of mature leaves; SLA-F: Specific leaf area of current year's fresh leaves; N_mass_-M: Nitrogen concentration of mature leaves; N_mass_-F: Nitrogen concentration of current year's fresh leaves; P_mass_-M: Phosphorus concentration of mature leaves; P_mass_-F: Phosphorus concentration of current year's fresh leaves; TDMC: Twig dry matter content; TWD: Twig wood density; SWD: Stem wood density. ^‡^: The significantly correlated relationships at the landscape scale that were robust relative to the choice of null models at the plot scale are in bold; r is the Pearson product-moment correlation coefficient, and p is the significance level. NS means not significant.

At the plot scale, the number of individuals per plot showed strong relationships with plant traits, but not with leaf P concentration in mature leaves (Table 1). Plant maximum height, LDMC in both mature and current year leaves, TDMC, TWD and SWD were positively correlated with species abundance. In contrast, MLA, SLA, leaf N concentrations in both mature and current year leaves, P concentration in current year leaves, and TLA were negatively correlated with species abundance. Total basal area was positively correlated with plant maximum height, LDMC and TWD, but negatively correlated with SLA and leaf N concentration in current year leaves. No significant correlation was found for other plant traits (Table 1).

### Shifts of plant trait-species abundance relationships over a range of single environmental properties

**Figure 1:**
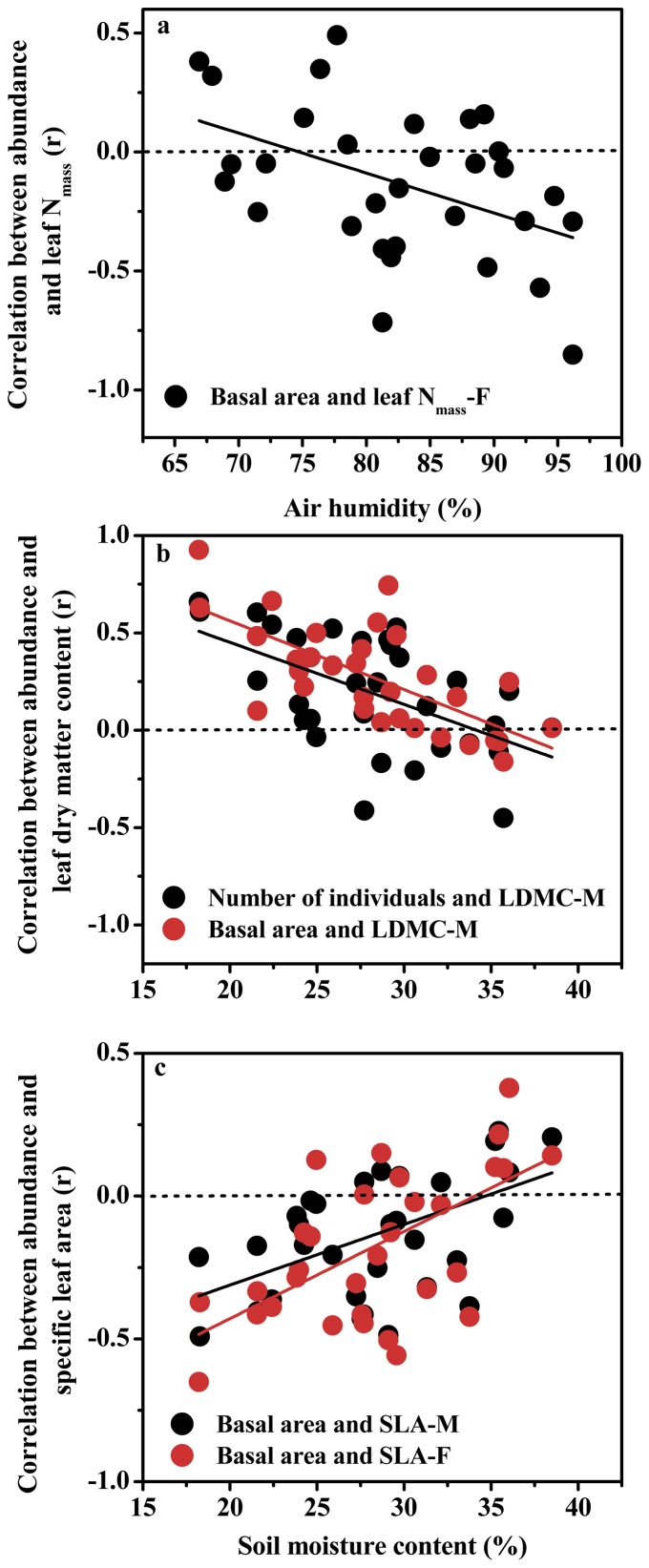
Shifting correlations between plant traits and species abundance at the plot scale over a range of air humidity (a) and soil moisture contents (b and c).

Most plant trait-species abundance relationships shifted in a range of each of the seven environmental properties. The correlation coefficients in the relationship between plant maximum height and each of the number of individuals (r = 0.74, p<0.001) and basal area (r = 0.66, p<0.001) were positively related with air humidity. In contrast, the r-values of the relationships between species abundance and each of leaf area (r = −0.41, p<0.05) and N concentration (r = −0.47, p<0.01) in current year leaves were negatively correlated with air humidity. In the range of air humidity, a significant direction reversal (from positive to negative) was found only in the relationship between basal area and N concentration in current year leaves (Fig. 1a), suggesting that abundant species in the wet habitat have lower N per leaf mass than those in the dry habitat.

There were significant negative correlations between soil moisture content and the correlation coefficients for LDMC in both mature (r = −0.70, p<0.001) and current year (r = −0.37, p<0.05) leaves vs. basal area, and for LDMC in mature leaves vs. the number of individuals (r = −0.55, p<0.01). In contrast, soil moisture content was positively correlated with the r-values in the relationships between SLA in both mature (r = 0.43, p<0.01) and current year (r = 0.53, p<0.01) leaves and basal area. In the soil moisture range, the relationship between LDMC in mature leaves and each of the number of individuals and basal area reversed from positive to negative (Fig. 1b), but the relationships between SLA in both mature and current year leaves and basal area changed from negative to positive (Fig. 1c). These contrasting reversing patterns in trait-abundance relationship demonstrate that species with large LDMC and low SLA are more abundant in the dry than in the wet habitats.

**Figure 2:**
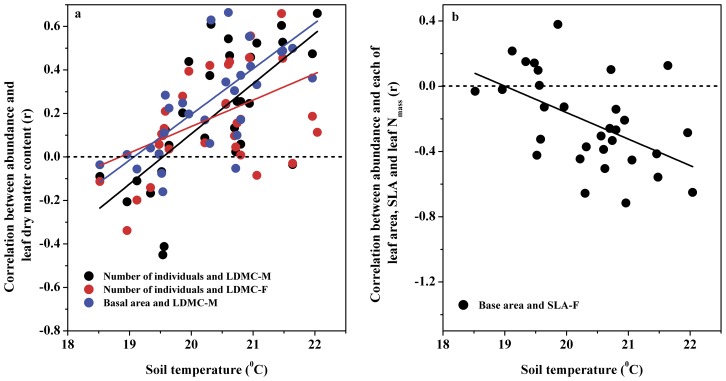
Shifting correlations between plant traits and species abundance at the plot scale over a range of soil temperatures.

Soil temperature was positively correlated with the r values for the relationships between LDMC in both mature and current year leaves and each of the number of individuals (mature leaves: r = 0.69, p<0.001; current year leaves: r = 0.43, p>0.05) and basal area (mature leaves: r = 0.71, p<0.001; current year leaves: r = 0.41, p>0.05). In contrast, the r values for the relationships between the number of individuals and each of MLA (r = −0.45, p<0.05) and leaf N concentration (r = −0.40, p<0.05) in mature leaves and TLA (r = −0.47, p<0.01), and the relationships between basal area and each of MLA (r = −0.38, p<0.05), leaf N concentration in mature leaves (r = −0.39, p<0.05) and SLA of current year leaves (r = −0.50, p<0.01) were negatively correlated with soil temperature. In the soil temperature range, the LDMC-abundance relationships reversed from negative to positive (Fig. 2a), whereas the relationships between basal area and SLA of current year leaves reversed from positive to negative (Fig. 2b). These reversed directions in the trait-abundance relationship across soil temperature indicate that species with high LDMC, but low SLA are more abundant in warm than in cold habitats.

**Figure 3:**
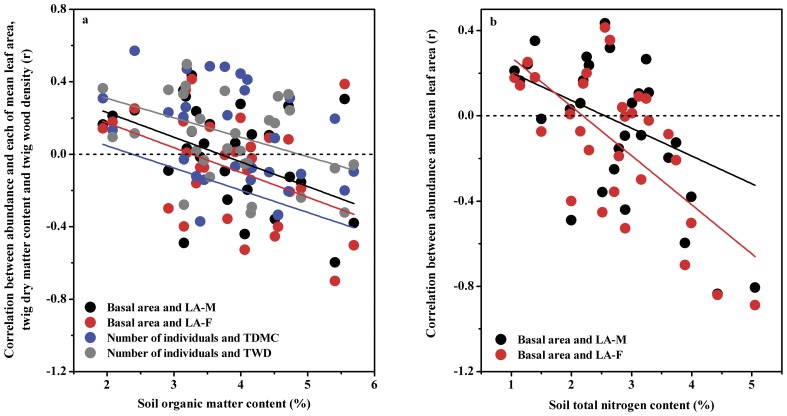
Shifting correlations between plant traits and species abundance at the plot scale over a range of soil organic matter (a), and soil total nitrogen content (b).

Soil organic matter content was negatively correlated with the correlation coefficients between species abundance and each of leaf area in both mature (r = −0.41, p<0.05) and current year (r = −0.41, p<0.05) leaves, TDMC (r = −0.38, p<0.05) and TWD (r = −0.41, p<0.05), but positively correlated with the r-values for the relationship between the number of individuals and SLA of the current year leaves (r = 0.37, p<0.05). In a range of soil organic matter content, the relationships between species abundance and each of leaf area in both mature and current year leaves, TDMC and TWD changed from positive to negative (Fig. 3a). This means that species with large MLA, TDMC and TWD are more abundant in soils with low than with high levels of organic matter content, and vice versa.

Soil N content was negatively correlated with the correlation coefficients between basal area and MLA in both mature (r = −0.41, p<0.05) and current year (r = −0.37, p<0.05) leaves. The relationships between basal area and MLA in both mature and current year leaves shifted from positive to negative over a range of soil N content (Fig. 3b), indicating that species with high values of leaf area were more abundant in soils with low N than those with high N contents. Soil P content was negatively correlated with the correlation coefficients between leaf P concentration in mature leaves and each of the number of individuals (r = −0.48, p<0.01) and basal area (r = −0.49, p<0.01). Since leaf P concentration in mature leaves did not correlate with species abundance, there was no significant reversal of trait-abundance relationship over a range of soil P content.

**Figure 4:**
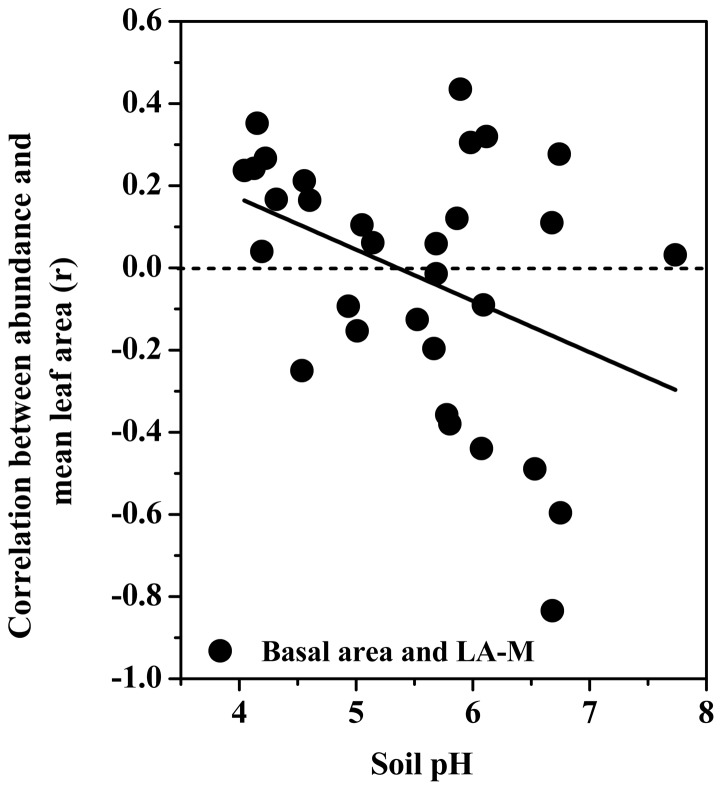
Shifting correlations between plant traits and species abundance at the plot scale over a range of soil pH.

Soil pH was positively correlated with the r-values for the relationships between LDMC in both mature and current year leaves and each of the number of individuals (mature leaves: r = 0.54, p<0.01; current year leaves: r = 0.55, p>0.01) and basal area (mature leaves: r = 0.59, p<0.001; current year leaves: r = 0.50, p>0.01). In contrast, soil pH was negatively correlated with the r-values in the relationships between MLA of mature leaves and each of the number of individuals (r = −0.45, p<0.05) and basal area (r = −0.39, p<0.05). Across a range of soil pH, the relationship between species abundance and MLA changed from positive to negative (Fig. 4). This shifting direction suggests that the most abundant species in soils with neutral pH have low values of MLA.

### Shifts of plant trait- species abundance relationship over a range of multivariate environmental properties

The first axis (PC 1) explained 38.9% of the overall variation and represented a general soil nutrient, acidity and temperature axis with positive loadings on soil organic matter (0.67), N (0.77) and P (0.51) contents, soil pH (0.75) and soil temperature (0.42), but with a negative loading on soil moisture (−0.46). The second axis (PC 2) explained 27.5% of the overall variation and represented an index of soil moisture and soil nutrient content with relatively large positive loadings on soil moisture (0.40), soil organic matter (0.29), soil N (0.29) and soil P (0.23), but with negative loadings on soil temperature (−0.55) and pH (−0.43).

**Figure 5:**
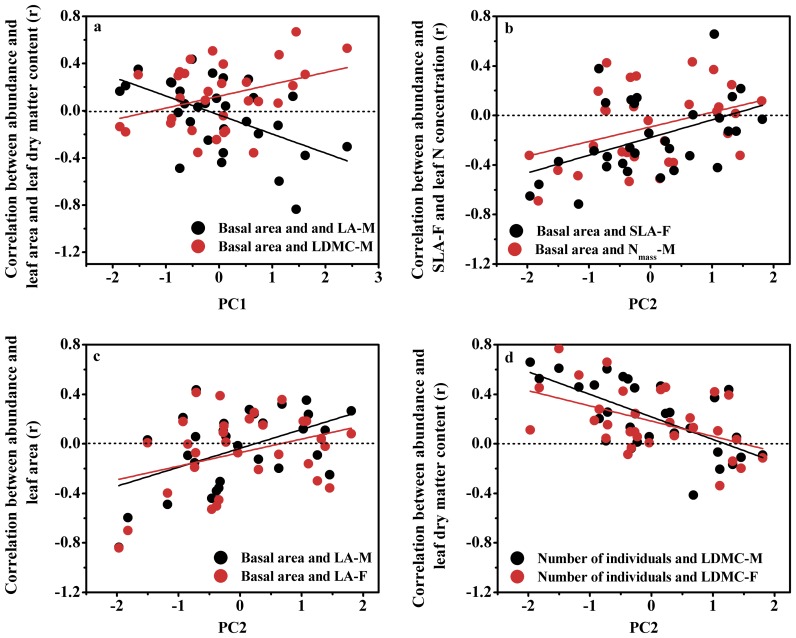
Shifting correlations between plant traits and species abundance at the plot scale over a range of major multivariate environmental properties.

The scores of PC 1 was positively correlated with the r-values for the relationship between MLA of mature leaves and basal area (r = 0.37, p<0.05), but negatively correlated with the r-values for the relationship between LDMC of mature leaves and basal area (r = −0.37, p<0.05). Along the first major axis, the relationship between species abundance and MLA reversed from positive to negative, but an opposite reversal was observed for the relationship between species abundance and LDMC (Fig. 5a).

The scores of PC 2 was positively correlated with the r-values for the relationships between the number of individuals and each of plant height (r = 0.47, p<0.01), SLA in current year leaves (r = 0.37, p<0.05), leaf N concentration in mature leaves (r = 0.36, p<0.05), and MLA of mature leaves (r = 0.38, p<0.01). Also, the scores of PC 2 was positively correlated with the r-values for the relationships between basal area and each of plant height (r = 0.50, p<0.01), SLA in current year leaves (r = 0.46, p<0.01), leaf N concentration in mature leaves (r = 0.38, p<0.05), and mean leaf area in both mature (r = 0.48, p<0.01) and current year (r = 0.35, p<0.01) leaves. In contrast, the scores of PC 2 was negatively correlated with the r-values for the relationships between the number of individuals and LDMC for both mature (r = −0.65, p<0.001) and current year (r = −0.48, p<0.01) leaves, as well as for the relationships between basal area and LDMC for both mature (r = −0.60, p<0.001) and current year (r = −0.36, p<0.05) leaves. Over the second major axis, the relationships between basal area and each of SLA (Fig. 5b) and MLA (Fig. 5c) in current year leaves, and leaf N concentration (Fig. 5b) and MLA (Fig. 5c) in mature leaves reversed from negative to positive. In contrast, the relationships between the number of individuals and LDMC in both mature and current year leaves reversed from positive to negative along the PC 2 axis (Fig. 5d).

## Discussion

### Plant trait-species abundance relationship

We found strong relationships between plant traits and species abundance among and within communities, supporting other lines of evidence that trait-based niche-assembly affects species abundance [15], [17], [19], [22], [23], [25], [27], [28], [35]. There are two main points that we would like to highlight in this section: (1) plant functional traits play a crucial role in affecting species abundance at both landscape and local scales, and (2) between mature and current year leaves, plant leaf trait-species abundance relationships are similar for physical traits (LDMC, MLA and SLA), but different for chemical traits (leaf N and P concentrations).

First, trait-based patterns of species abundance can be attributed to the functional strategy of a species. For example, plant potential maximum height, wood density, leaf size and leaf N concentration are associated with ecological strategies such as resource acquisition and retention that are related to the successional status of a species. Species with high wood density, low leaf size and N concentration can retain nutrients for longer periods of time [39]–[40]. Species with high potential maximum height have advantages in light capture. Therefore, the combination of nutrient acquisition and the potential to reach a greater maximum height can be thought of as strategies for late-successional species [19], [39]. In this study, due to absence of large-scale disturbances in the past several decades [41], the vegetation has proceeded to the mature stage during secondary forest succession. The most abundant species are late-successional, characterized by the use of the ‘conservative consumption’ strategy of resource use [45]. Consequently, at the landscape scale, species with high maximum height and wood density, and low MLA, TLA and leaf N concentration are more abundant than other species across the five studied sites (i.e., the regional species pool). In contrast, early successional species are found mainly at the young shrub stage in the DQ site, where local disturbances such as selective logging happen periodically. The species composition is often characterized by resprouting evergreen broadleaf and deciduous plants [41]. Even in this vegetation type, the most common resprouting evergreen species (e.g., *Schima superba*) usually have the same attributes in acquiring and retaining resources compared to the late-successional species, and deciduous species only take up resources in the way of ‘resource spending’ [45]. Therefore, the most abundant species across sites are characterized by the ecological strategy of ‘conservative consumption’. This is consistent with the observation that the most abundant species use the late-successional strategy at the Jasper Ridge Biological Preserve, California, USA [19].

At the plot scale, strong relationships between species abundance and each of plant maximum height, wood density, leaf size, and leaf N concentration existed. Moreover, plant trait-species abundance relationships suggest that species with high LDMC and TDMC, but low SLA and leaf P concentration are more abundant than coexisting species in a given community. The relatively high LDMC and low SLA and leaf P concentration are associated with plant resource use strategies of ‘conservative consumption’ [33], [46]. As discussed above, relative to the less abundant species in the studied sites, the functional strategies of the most abundant species are associated with a higher level of resource retention. As a result, species abundance should be correlated with the functional traits of the species.

Second, for physical traits (LDMC, MLA and SLA), plant leaf trait-species abundance relationships were consistent between mature and current year leaves at both the landscape and plot scale. However, in relation to species abundance, leaf chemical traits (leaf N and P concentrations) showed different patterns between mature and current year leaves at the plot scale (Table 1). Functionally, leaf traits are variable as they change with leaf longevity, and variation in leaf longevity is associated with a wide array of factors such as the physiology, anatomy and ecology of plants [31], [33]. In this study, leaf chemical trait-species abundance relationships differ between mature and current year leaves. Particularly, the variable relationships between leaf chemical trait-species abundance exist at the plot scale only. This suggests that variations in species-based leaf chemical traits are greater within than among plots, which is why leaf chemical trait-species abundance relationships differ between leaf ages within a plot, instead of between plots. The underlying mechanisms for varied leaf chemical trait-species abundance relationships between mature and current year leaves within a plot might 1) be related to the idea that chemical evenness (over dispersion) among species may reduce antagonistic interactions with neighbors [12], [25]; such antagonistic interactions maybe mediated by natural enemies [47], and 2) reflect within-site niche differentiation [15], [18], [27], [34]. In contrast, physical leaf traits that exhibit less variation in the trait-species abundance relationship may be related to the species tolerances to microsite or environmental changes [47]. The mechanism underlying this pattern deserves further study in the future.

### Shifts in plant trait-species abundance relationship with changes of environmental properties

In accordance with our predictions, most plant trait-species abundance relationships shifted over a range of both single and multivariate environmental properties. In relation to air humidity, species with low leaf N concentration are more abundant in the wet than in the dry habitats (Fig. 1a). Across a range of soil moisture content, species with high SLA but low LDMC are more abundant in the wet than in the dry habitats (Fig. 1b, 1c). It is fairly well-understood that species from drier habitats have generally higher leaf N per mass than those from wetter sites [37]. The expected reversed pattern in SLA (lower at drier sites) was supported by the data. Anatomically, higher N_mass_ could reflect a larger fraction of volume of mesophyll cells within the leaf [37]. As a response to the stronger average irradiance in drier habitats, species is expected to have low mean SLA and high LDMC. On the other hand, due to the high humidity in wetter habitats (e.g., the ravine area), species tends to have higher values of SLA and low LDMC.

In relation to soil temperature, species with high LDMC and low SLA are more abundant in warm than in cool habitats (Fig. 2). It is well understood that warmer habitats are generally more dried than cool habitats if humidity is similar between them. In this study, the principle component analysis demonstrated that soil temperature loads a positive but soil moisture loads a negative score on the PC 1 axis. This means that warm habitats associate with a drier soil condition among the studied plots. As such, the most abundant plants in warm habitats tend to exhibit the same traits as plants from dry habitats.

Compared to habitats with high soil organic matter and N contents, the most abundant species in nutrient-poor habitats are characterized by a large leaf area (Fig. 3). This pattern is not consistent with the widely acknowledged hypothesis that plants with conservative leaf strategies usually inhabit nutrient-poor soils with small leaf size [31], [33], [37]. In this study, the most abundant plants found in nutrient-poor habitats are usually deciduous species [42]. Since deciduous plants takes up nutrient generally in the way of ‘resource spending’ [45], it is understandable that leaf size is large for the most abundant plants in nutrient-poor habitats. In addition to the small leaf size, the most abundant species on nutrient-rich soils also have lower TDMC and TWD, relative to nutrient-poor soils (Fig. 3a). This is due to that plants in nutrient-rich soils tend to grow very tall [31]. In terms of trade-offs between plant growth and mechanical support in biomass allocation [31], [33], plants grow rapidly at the expense of mechanical construction, and vice versa [48]. Therefore, with high growth rates, the most abundant species on nutrient-rich soils tends to reduce wood density and TDMC [33], [49].

Soil pH could influence plant growth, because it affects the availability of needed nutrients [31], [50]. Soil pH in our studied plots ranged from 3.5 to 7.8. Since proton concentration is high in acidic soils, nutrients (e.g., N) may be freed quickly to increase N availability [51]. In this case, plants may follow the strategy of ‘resource-spending’ in acidic soils, and thus display high values of leaf area. Interestingly, shifting patterns in plant trait-species abundance relationship along a range of environmental properties are consistent between single and multivariate environmental properties. In this study, the first axis represented multivariate space for soil organic matter and N contents and acidity. Along this axis, species with greater LDMC but lower MLA are more abundant in habitats with nutrient-rich and acidic soils than in habitats with nutrient-poor and neutral (pH) soils. The second axis represented an index of soil moisture and nutrient contents. Along this multivariate space, the most abundant species in wet and nutrient-rich habitats are characterized by larger SLA but smaller LDMC, than species in dry and nutrient-poor habitats.

## Conclusions

In conclusion, strong relationships between plant trait and species abundance were established among and within communities. The existence of trait-abundance correlations provides evidence for non-neutral processes that affect the abundance and rarity of woody plant species in the studied evergreen subtropical forests in eastern China. The significant shifts in plant trait-species abundance relationships over a range of single and multivariate environmental properties suggest a strong environmental filtering process that influences species abundance.
